# Epidemiological Surveillance of Birth Defects Compatible with Thalidomide Embryopathy in Brazil

**DOI:** 10.1371/journal.pone.0021735

**Published:** 2011-07-06

**Authors:** Fernanda Sales Luiz Vianna, Jorge S. Lopez-Camelo, Júlio César Louguercio Leite, Maria Teresa Vieira Sanseverino, Maria da Graça Dutra, Eduardo E. Castilla, Lavínia Schüler-Faccini

**Affiliations:** 1 INAGEMP (Instituto Nacional de Genética Médica Populacional) at Teratogen Information Service, Medical Genetics Service, Hospital de Clínicas de Porto Alegre, Porto Alegre, Brazil; 2 INAGEMP at Genetics Department, Universidade Federal do Rio Grande do Sul, Porto Alegre, Brazil; 3 INAGEMP at ECLAMC (Latin-American Collaborative Study of Congenital Malformations) in IMBICE: Instituto Multidisciplinario de Biologia Celular, La Plata, Argentina; 4 INAGEMP at ECLAMC in CEMIC: Centro de Educación Médica e Investigación Clínica, Buenos Aires, Argentina; 5 INAGEMP at ECLAMC in Laboratório de Epidemiologia de Malformações Congênitas, Instituto Oswaldo Cruz, FIOCRUZ, Rio de Janeiro, Brazil; University of Ottawa, Canada

## Abstract

The thalidomide tragedy of the 1960s resulted in thousands of children being born with severe limb reduction defects (LRD), among other malformations. In Brazil, there are still babies born with thalidomide embryopathy (TE) because of leprosy prevalence, availability of thalidomide, and deficiencies in the control of drug dispensation. Our objective was to implement a system of proactive surveillance to identify birth defects compatible with TE. Along one year, newborns with LRD were assessed in the Brazilian hospitals participating in the Latin-American Collaborative Study of Congenital Malformations (ECLAMC). A phenotype of LRD called thalidomide embryopathy phenotype (TEP) was established for surveillance. Children with TEP born between the years 2000–2008 were monitored, and during the 2007–2008 period we clinically investigated in greater detail all cases with TEP (proactive period). The period from 1982 to 1999 was defined as the baseline period for the cumulative sum statistics. The frequency of TEP during the surveillance period, at 3.10/10,000 births (CI 95%: 2.50–3.70), was significantly higher than that observed in the baseline period (1.92/10,000 births; CI 95%: 1.60–2.20), and not uniformly distributed across different Brazilian regions. During the proactive surveillance (2007–2008), two cases of suspected TE were identified, although the two mothers had denied the use of the drug during pregnancy. Our results suggest that TEP has probably increased in recent years, which coincides with the period of greater thalidomide availability. Our proactive surveillance identified two newborns with suspected TE, proving to be a sensitive tool to detect TE. The high frequency of leprosy and the large use of thalidomide reinforce the need for a continuous monitoring of TEP across Brazil.

## Introduction

Thalidomide was first synthesized in 1954 in Western Germany and introduced to the market in 1956. Subsequently it was licensed in a further 46 countries worldwide, including Brazil [1], [2]. Limited studies in animals had suggested that thalidomide was not toxic, which indicated it was a safe sedative when compared to barbiturates [3]. However, a great number of babies with congenital defects, especially limb reduction, were born at the beginning of the 1960s, something that was promptly detected by “alert practitioners” [4], [5], [6]. These malformations were characterized by defects in the development of the long bones of the limbs, with hands and feet varying between normal and rudimentary. Besides limb reduction defects (LRD), associated malformations were also documented, such as anotia, microtia, anophthalmia, and microphthalmia, as well as cardiac, genitourinary and gastrointestinal anomalies [7]. At the end of 1961 [5], [6] Lenz in Germany, and McBride in Australia [4] suggested a possible correlation between these congenital defects and the use of thalidomide during pregnancy. The drug was removed from the market in Germany and in several other countries between 1961 and 1962, by which time some 10,000 child victims of thalidomide had already been born worldwide [2].

A few years later, Sheskin in 1965 [8] reported the effectiveness of thalidomide in the treatment of erythema nodosum leprosum (ENL), an inflammatory condition resulting from leprosy. He prescribed this drug to a leprosy patient as a sedative, and observed the complete improvement of symptoms and skin lesions within three days. The proven efficacy of the drug for this indication [9] increased the general interest in the drug's therapeutic potential for other conditions, especially after its anti-inflammatory, immunomodulating and anti-angiogenic proprieties were recognized [10], [11], [12].

Based on the knowledge of these properties, several clinical trials set out to demonstrate the effectiveness of the drug for the treatment of various medical conditions. In 1998, thalidomide was approved by the US FDA for the treatment of ENL and later, in 2006, for the treatment of multiple myeloma, under strict restrictions to prevent exposure *in utero* [13]. Presently, the use of thalidomide is approved in many countries for the treatment mainly of ENL, skin diseases, and several types of cancer.

In Brazil, thalidomide has always been available in the regions with endemic leprosy. In 1965 Brazil approved its use for the treatment of ENL [14]. This continuous commercialization plus its high use due to the prevalence of leprosy and inefficient drug control measures gave way to the appearance of new cases of thalidomide embryopathy (TE) between the 1970s and 1990s [15]. Following these reports, a more restrictive regulation was created for thalidomide use and prescription in Brazil [16]. Nevertheless, three new individuals with thalidomide syndrome were reported after that [17].

Besides being employed in the treatment of ENL since 1965, thalidomide has been available for use in Brazil since the end of the 1990s for the treatment of multiple myeloma, graft-versus-host reaction, systemic lupus erythematosus, and ulcerations related to the acquired immunodeficiency syndrome (AIDS), among other diseases, as long as the purpose of prescription in these situations is duly documented. Leprosy is definitely the main disease to which thalidomide has been prescribed. Brazil, with a population of 190 million inhabitants is one of the leading countries in number of leprosy cases the world, with an overall estimated prevalence of 5/10,000. However, regional prevalences are quite dissimilar, ranging from less than 1/10,000 in South Brazil to 7/10,000 in North and Northeast [18].

The drug is not commercially available being distributed only through specific programs of the Ministry of Health, and dispensed following explicit and rigid rules. However, the recent discovery of babies with thalidomide embryopathy (TE) [17] raises questions as to the effectiveness of the restricted distribution system with respect to prevention of pregnancy exposures. Thus, the objective of the present study was to perform a proactive surveillance of thalidomide embryopathy phenotype (TEP) using an established system for monitoring birth defects in Latin America.

## Results

During the baseline period (1982–1999), of the 793,177 births examined 152 newborns presented TEP. The BPR observed was 1.92/10,000 births (CI 95%: 1.60–2.20), and no significant difference between geographical regions was detected (Table 1).

**Table 1:** Number of newborns with TEP and BPR in the period of 1982–1999 by Poisson distribution.

Region	TEP	Births	BPR	CI 95%
**Northeast**	22	155,784	1.41	0.90–2.10
**Southeast**	94	423,261	2.22	1.80–2.80
**South**	36	214,132	1.68	1.20–2.30
**Total**	**152**	**793,177**	**1.92**	**1.60–2.20**

Footnote: TEP: thalidomide embryopathy phenotype; BPR: birth prevalence rate; BPR per ten thousand births.

During the surveillance period (2000–2008), of 352,037 births assessed, 109 newborns fitted our definition of TEP, which generated a BPR of 3.10/10,000 births (CI 95%: 2.50–3.70) (Table 2). Overall, BPR was higher than that observed for the baseline period. Among the regions assessed, the southeast presented the highest BPR in the surveillance period (Tables 1 and 2). On the other hand, the south region presented the lowest BPR compared to other regions during the surveillance period, being similar during baseline and surveillance periods (Table 1 and 2).

**Table 2:** Number of newborns with TEP and BPR, per year and geographical region, in the period of 2000–2008 by Poisson distribution.

	Brazil				Northeast				Southeast				South			
Year	TEP	Births	BPR	CI 95%	TEP	Births	BPR	CI 95%	TEP	Births	BPR	CI 95%	TEP	Births	BPR	CI 95%
**2000**	11	40104	2.74	1.40–4.90	**-**	**-**	**-**	**-**	6	12047	5.00	1.80–10.80	5	28057	1.80	0.60–4.20
**2001**	10	38742	2.50	1.20–4.70	0	5088	0.00	0.00–10.2	8	11876	6.74	2.90–13.30	2	21778	0.92	0.10–3.30
**2002**	10	40231	2.49	1.20–4.60	0	6617	0.00	0.00–7.8	8	12197	6.56	280–12.80	2	21417	0.93	0.10–3.40
**2003**	10	42061	2.38	1.10–4.40	0	10171	0.00	0.00–4.90	4	11466	3.50	0.90–8.90	6	20424	2.94	1.10–6.40
**2004**	11	42581	2.58	1.30–4.60	2	9733	2.10	0.20–7.40	5	11316	4.42	1.40–10.30	3	21532	1.40	0.30–4.10
**2005**	16	37518	4.26	2.40–6.90	7	10196	6.90	2.80–14.10	5	10909	4.60	1.50–10.70	5	16413	3.05	1.00–7.10
**2006**	15	38298	3.92	2.20–6.50	6	9341	6.42	2.30–14.00	4	13021	3.10	0.80–7.90	5	15936	3.24	1.00–7.30
**2007**	16	38281	4.18	2.40–6.80	2	8627	2.32	0.30–8.40	13	14658	8.90	4.70–13.20	1	14996	0.70	0.10–3.70
**2008**	10	34221	2.92	1.40–5.40	5	8920	5.60	1.80–13.10	3	13192	2.30	0.50–6.60	2	12109	1.65	0.20–6.00
**Total**	**109**	**352037**	**3.10**	**2.50–3.70**	**22**	**68693**	**3.20**	**2.00–4.80**	**56**	**110682**	**5.10** *	**3.80–6.80**	**31**	**172662**	**1.80** *	**1.20–2.50**

Footnote: TEP: thalidomide embryopathy phenotype; BPR: birth prevalence rate; CI 95%: confidence interval of 95%.

*p<0.05.

**-**: year without monitoring by ECLAMC. BPR per 10 thousand births.

It is noteworthy also that during the surveillance period the overall Brazilian frequency of LRD (8.50, CI 95%: 7.50–9.80) was significantly higher than that registered by ECLAMC hospitals in other Latin American countries (6.83, CI 95%: 6.40–7.30; data not shown).

### Cumulative Sum Analysis (CUSUM)

**Figure 1:**
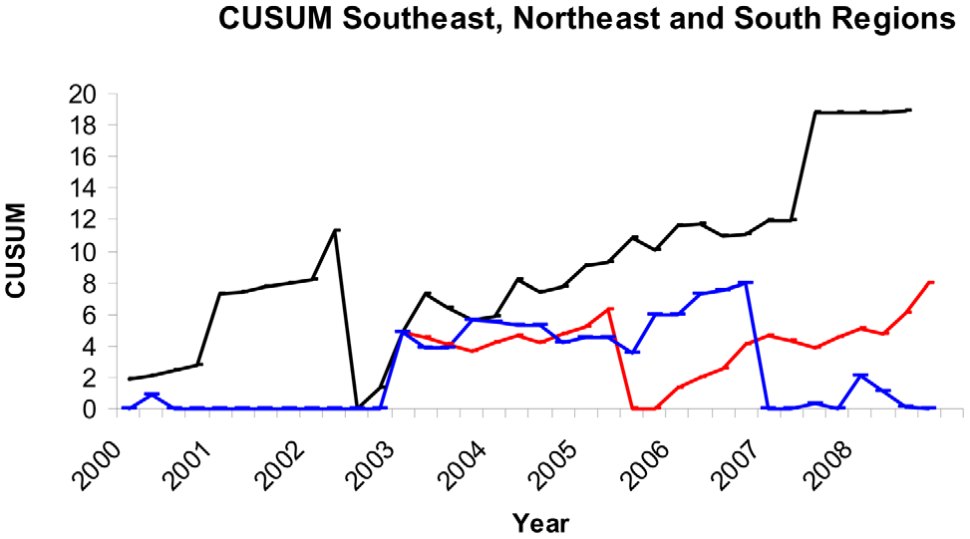
CUSUM analysis done for thalidomide embryopathy phenotype for southeast (black line), northeast (red line), and south (blue line) Brazilian regions. Parameters: Southeast region (K = 0.8 H = 10 ARL = 518 OOCARL = 44.4); northeast region (K = 0.4 H = 5.6 ARL = 473 OOCARL = 44.2); and south region (K = 1 H = 8 ARL = 462.1 OOCARL = 36.4).

During the surveillance period (2000–2008), increases in the frequencies of TEP were observed in all Brazilian geographical regions (Figure 1), but only in the southeast and northeast regions were the alarms confirmed.

### Proactive Surveillance

After detailed clinical evaluation (photographs, radiographs and a structured questionnaire) of the 96 babies born with LRD during the proactive surveillance period (March 2007 to February 2008), 16 neonates were classified as affected by TEP. Of these, seven cases were considered inconclusive because of lack of information and seven were incompatibles. Thus, two infants had phenotypes suggestive of thalidomide use during pregnancy and had not received a diagnosis of any other type of syndrome. However, the use of thalidomide was not confirmed by their mothers when specifically questioned. These two cases are described in detail below. For the second year of surveillance clinical evaluation is not yet complete.

#### Case 1

Female born in July 2007 in the northeast of Brazil; birth weight 2,360 g. The mother was 29 years old with no family history of malformations and with no pregnancy exposure. Upper limbs: bilateral intercalary defect; absent left humerus, hypoplastic radius and ulna; hypoplastic left hand with two rudimentary digits; hypoplastic right radius and ulna, hypoplastic hand with two rudimentary fingers; both lower limbs normal. Besides the LRD, this newborn presented unilateral cleft lip. The electrocardiogram was normal.

#### Case 2

Male born in October 2007, in the same hospital as case 1; birth weight 2,405 g. The third son of a 34-year-old mother with no family history of malformations or genetic diseases; his mother had no exposure to thalidomide during pregnancy. Tetramelic amelia, bilateral microtia, elongated nose root, moderate retromicrognathia, and left cryptorchidism. Electrocardiogram was normal.

## Discussion

Generic thalidomide is produced in Brazil by just one laboratory, under supervision of the Ministry of Health. Around four million tablets of thalidomide are distributed yearly, by specific government programs, mostly for the treatment of ENL. Until 2010, there was no information about the exact destination of these tablets. This lack of information can be accountable for the recent occurrence of cases of thalidomide syndrome. From 2011, a new legislation for thalidomide dispensing was implemented in Brazil with a strong control of to whom this drug is being prescribed [27]. However, we know that in Brazil around 24,000 cases of multibacilar leprosy are yearly diagnosed. From these, 30% to 50% will present ENL. From this estimation, approximately 10,000 individuals are possible users of thalidomide.

The assessment of TEP during the baseline period enabled the establishment of a Brazilian BPR for phenotypes compatible with this syndrome, permitting the detection of increases in the frequency of TEP through the CUSUM methodology.

There are no references with which to compare the rates of a sentinel phenotype as described here; however, during the surveillance period, increases in the BPRs of TEP were observed, corroborating the thalidomide distribution pattern from 2000. The differences observed in the TEP rate between the different regions of Brazil are in accordance with the distribution of leprosy across the country [20]. In the south region, TEP was less frequent than in other regions, and so was leprosy prevalence. Furthermore, no differences between the two periods were detected. The southeast region presented the highest BPR, although this result may have been biased by data collected in specialized maternities belonging to ECLAMC, which have a higher rate of birth defects, especially from cases referred to them after prenatal diagnosis. The alarms detected in the CUSUM analysis followed the same pattern.

This significant increase in the frequency of LRD could have been biased by the improvement in prenatal diagnosis and derivation of those cases with fetal anomalies to referral hospitals participants of ECLAMC. Excluding tertiary hospitals in both periods, the increased rate of TEP is not observed (baseline period: 1.59, 95% CI :1.30–1.87; period of surveillance: 2.18, 95% CI 1.64–2.71).

Our proactive surveillance led to the identification of two cases compatible with TE, although maternal use of thalidomide could not be proven. However, the availability of this information often depends on individual conditions, such as maternal memory and fear of social prejudice due to of the use of a medication that is contraindicated during pregnancy. Moreover, there is the possibility of self-medication, which is a habitual behavior among the Brazilian population and lies behind the unadvised use of several drugs during pregnancy. This is a problem observed not only with thalidomide but also with other drugs with teratogenic potential. In three recent clinically characteristic cases of embryopathy recorded in Brazil [17], maternal interview was negative for the use of thalidomide.

It is important to point out that thalidomide is not the only etiological factor for the phenotypes that we included as suggestive of TE. Syndromes whose characteristics are similar to those of TE include: Roberts syndrome, Holt-Oram syndrome, Fanconi's pancytopenia, radial aplasia-thrombocytopenia (TAR), among others syndromes, as well and Femur-Fibula-Ulna complex [1], [7], besides unspecified developmental conditions.

One limitation of the present surveillance is that the main endemic areas of leprosy in Brazil are located in rural regions, especially in the north and center-west regions, where many births take place outside hospital settings and where coverage and monitoring by ECLAMC is not present. In any case, the percentage of coverage of births is also a limiting factor in surveillance systems.

Yang et al. [22] evaluated the ability of monitoring systems to detect TE alarms and suggested that the surveillance of all LRD is insufficient for the detection of this type of embryopathy. They support the notion that an impracticable surveillance time would be necessary when the rate of exposure to thalidomide is very low, even when monitoring bilateral intercalary and preaxial defects (or only intercalary, which are the defects most frequently associated with TE). In the present study, however, local accessibility to thalidomide was high, and we proposed the cumulative sum methodology (CUSUM) for the detection of increases in the frequency of alarms, since it is a method which is faster at detecting changes in prevalences than the Poisson methodology [22]. The TE surveillance system presented herein is highly sensitive because all the LRD described in the syndrome are included, but the system has low specificity because it groups different types of LRD not related to TE. This bias was controlled by the direct assessment of all the TEP reported cases.

We believe that the surveillance protocol presented here is feasible and sensitive to immediately detect new cases of thalidomide embryopathy cases. This surveillance will be maintained at population level in Brazil through official birth certificates registry, which includes mandatory description of birth defects.

The present paper should be considered as an alert toward the prevention of an announced tragedy mainly in developing countries. It also points the necessity to develop more precise and controlled national systems that permit to identify and to prevent the abuse observed in prescription of drugs widely known as teratogens as well as the necessity to improve the diagnosis procedures in children with complex limb anomalies.

## Methods

### Ethics Statement

The surveillance was carried out on the data generated by ECLAMC (The Latin-American Collaborative Study of Congenital Malformations). ECLAMC is a program for the clinical and epidemiological investigation of risk factors in the etiology of congenital anomalies in Latin-American hospitals, using a case-control methodological approach already described elsewhere [19]. ECLAMC has been performing quarterly surveillance of TE since 1982. Participation of all Brazilian institutions active in the ECLAMC network was approved by their local ethics committees, and included the signing of a consent term for the publication of data. This investigation was approved by the ethics committee of CEMIC (Centro de Educación Médica e Investigaciones Clínicas), Buenos Aires, Argentina (IRB-000001745, IORG-0001315).

### Baseline Period and Surveillance

Two periods were examined: a baseline period (1982–1999) and a surveillance period (2000–2008); from 2007 onwards we clinically investigated in closer detail all TEP cases (proactive surveillance).

During all periods, 56 Brazilian hospitals were included in the analysis covering 1,145,214 births, representing 23.52% of 4,868,490 births in ECLAMC surveilled from nine different Countries in Latin America.

We analyzed the frequency of variation within hospitals by comparing the frequency during the surveillance period with the frequency of the baseline period within each hospital. For this, we calculated the observed and expected and used the Z test according to the Poisson distribution.

The period between the years 1982 and 1999 was established as a baseline period for TEP surveillance since the availability of thalidomide is suspected to have increased after 2000 due to the expansion in clinical indications for its prescription authorized by the Brazilian Health Ministry. Geographical regions were considered too, taking into account the differential prevalence of leprosy in Brazil [20]. The Poisson distribution, with a confidence interval of 95% was used to estimate birth prevalence rate (BPR).

The CUSUM methodology [21] was used to detect possible increases in TEP frequency after 2000. CUSUM has already been widely used for birth defects surveillance [22], [23], [24], being able to detect variations of TEP from the BPR of the baseline period by the sum of differences between the number of cases occurring during the surveillance period and a reference value obtained from the baseline period. The false alarm rate was set to one in 500 months (average run length (ARL)  = 500).

Detailed clinical proactive surveillance was conducted from March 2007 to February 2008 with records of newborns from 33 Brazilian hospitals participating in ECLAMC. All newborns with limb reduction defects were assessed and classified according to the type of limb defect and compatibility with TEP.

### Thalidomide Embryopathy Phenotype (TEP)

Newborns with preaxial and bilateral intercalary LRD came into this category, as these are already well established in the literature as TE sentinel phenotypes [15], [22]. Also, individuals that presented amelia were included, since it is a defect frequently observed in TE [25]. However, defects were included regardless of laterality, since this is information that can be lost during registration. This surveillance sentinel phenotype was called TEP.

The following LRD classification, adapted from [26], was adopted:

- Amelia: complete absence of one or more limbs;
- Defect of intercalary transverse limb reduction: absence or severe hypoplasia of proximal limb parts (humerus, femur, radius, ulna, tibia, and fibula, also in combination) with normal or approximately normal hands and feet. This group included phocomelia.
- Defect of preaxial longitudinal limb reduction: total or partial absence of thumbs, first metacarpus or radius; or hallux, first metatarsus, and tibia;

Limb defects that did not fit into any of these classifications were not included in the analysis.

Whenever TEP was identified, photographs and radiographs were requested, and a detailed maternal interview was conducted with the aim of investigating a possible exposure to thalidomide, including questions such as the use of medications during pregnancy, family history of congenital abnormalities, abortion attempt, and a diagnosis (in herself or in a close relative) of leprosy or other disease for which thalidomide use is approved in Brazil.

All newborns with TEP were also assessed according to compatibility with TE based on the following criteria:

- Presence or absence of congenital defects described in the literature relating to TE.
- Presence or absence of another known etiological syndrome that shows the same defects.

Maternal history of thalidomide use or of another associated risk factor: patient or close relative affected by leprosy, or another disease for which thalidomide has been employed: multiple myeloma, AIDS, lupus, graft-host reaction.
